# Supplementary material for: Unraveling the mechanism of small molecule induced activation of Staphylococcus aureus signal peptidase IB
Source: Commun Biol. 2024 Jul 24;7:895. doi: 10.1038/s42003-024-06575-x (PMC11266668; doi:10.1038/s42003-024-06575-x)
Supplement: Supplementary file 2 — Supporting Information [file 42003_2024_6575_MOESM2_ESM.pdf]

Supporting Information

Unraveling the mechanism of small molecule induced activation of *Staphylococcus aureus* signal peptidase IB

Shu-Yu Chen*[a,d], Michaela K. Fiedler*[b], Thomas F. Gronauer[b], Olesia Omelko[b], Marie-Kristin von Wrisberg[c], Tao Wang[b], Sabine Schneider[c], Stephan A. Sieber [+b], Martin Zacharias[+d]

Affiliations

a Department of Chemistry and Applied Biosciences, ETH Zurich, Vladimir-Prelog-Weg 2, Zurich, 8093, Switzerland

b TUM School of Natural Sciences, Department Biosciences, Chair of Organic Chemistry II, Center for Functional Protein Assemblies (CPA), Technical University Munich (TUM), Ernst-Otto-Fischer Str. 8, Garching, 85748, Germany

c Department of Chemistry, Ludwig-Maximilians University Munich (LMU), Butenandtstr. 5-13, 81377 Munich, Germany

d TUM School of Natural Sciences, Department Biosciences, Theoretical Biophysics (T38), Center for Functional Protein Assemblies (CPA), Technical University Munich (TUM), Ernst-Otto-Fischer Str. 8, Garching, 85748, Germany

* contributed equally to this work

[+] corresponding authors (stephan.sieber@tum.de, zacharias@tum.de)

Table of Content

Supplementary Figures S1 – S15 3
Supplementary Tables S1 – S5 13
Supporting References 15

Supplementary Figures S1 – S15

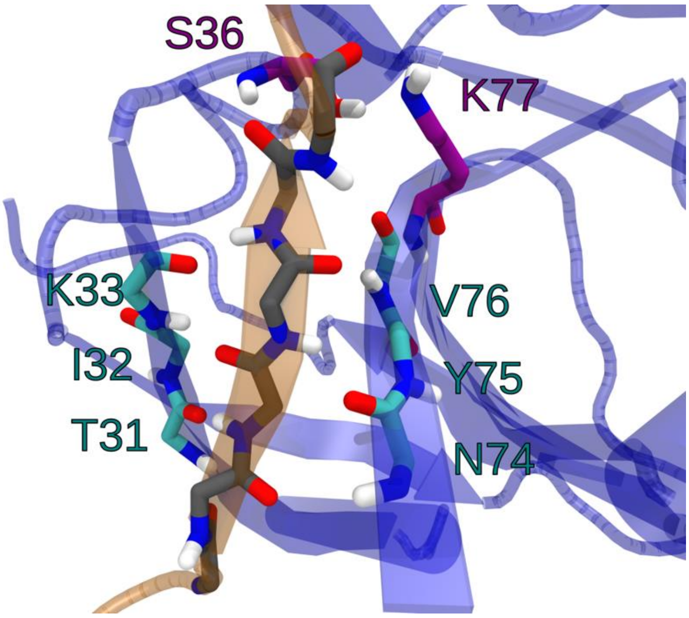

Figure S1: The hybrid β-sheet near the catalytic residues S36 and K77 (magenta) between the substrate (orange) and the enzyme (blue). Only the backbone atoms of the hybrid β-sheet are shown for better visibility.

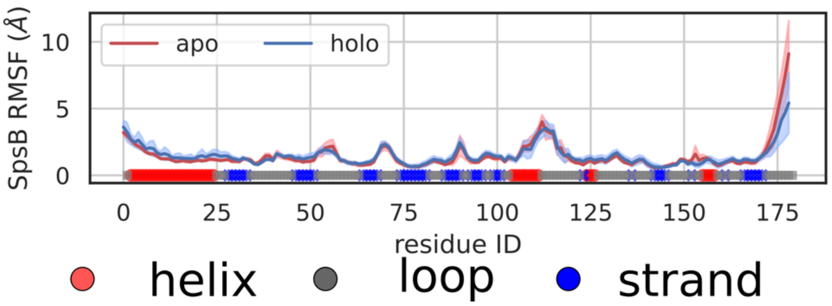

Figure S2. Stability analysis of SpsB with residue-wise root-mean-square-fluctuation (RMSF) in apo-form (red) and holo-form (blue). The corresponding secondary structure of each residue is indicated by the colors at the bottom.

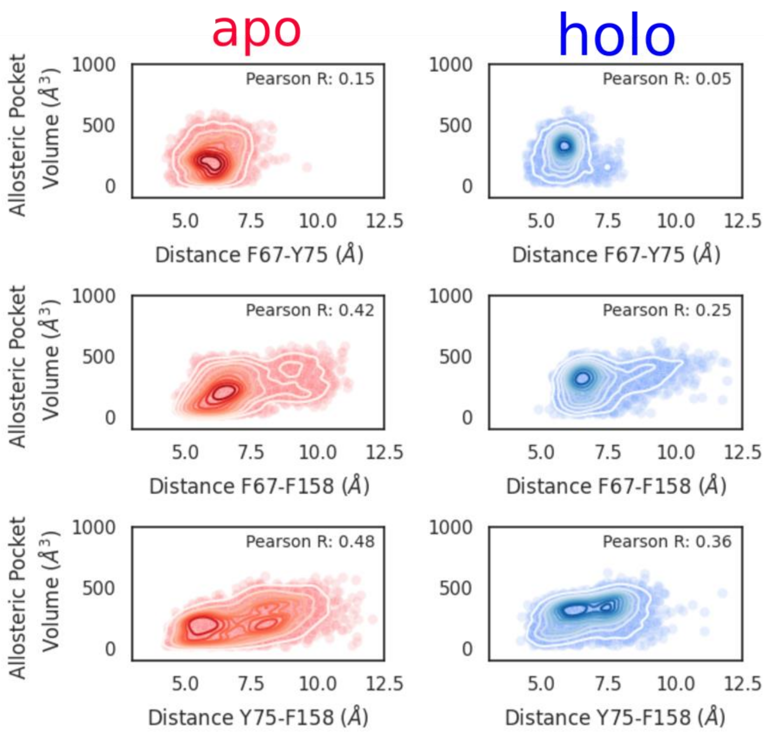

Figure S3. Correlation between the F67-Y75-F158 distances and the volume of the allosteric pocket in the apo-form (red) and holo-form (blue). The number on the upper right shows the Pearson correlation.

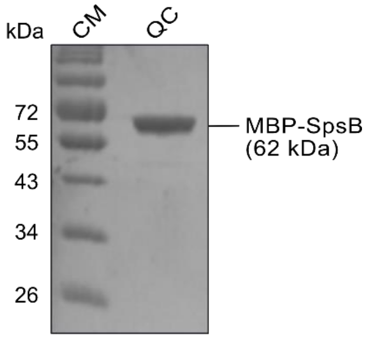

Figure S4: SDS Page gel of purified MBP-tagged extracellular domain of SpsB for binding site identification studies. CM = Coomassie marker, QC = quality control of purification. MBP-tagged extracellular SpsB domain = 62033 Da.

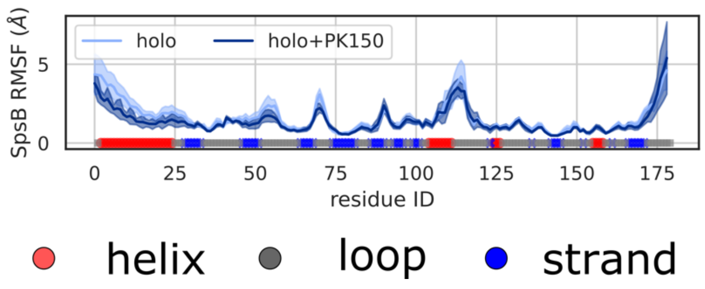

Figure S5: Fluctuation of the enzyme in the holo (light blue), and holo+**PK150** (dark blue) states indicated by residue-wise root-mean-square fluctuation (RMSF). The corresponding secondary structure of each residue is indicated by the color at the bottom.

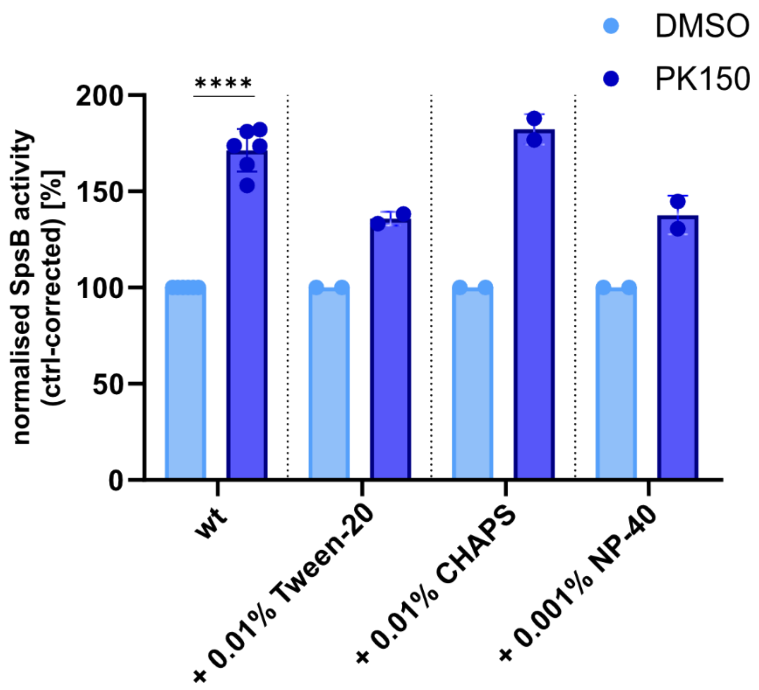

Figure S6: SpsB activity assay: PK150-induced (10 µM) cleavage of the FRET substrate by membrane-bound wt SpsB (50 µg ml^{-1} total membrane protein concentration) with or without addition of detergents (<cmc, below critical micellar concentration; (0.01% Tween[1] and 0.001% NP-40[2,3], 0.1% CHAPS = 1.6 mM < (5.4 – 11) mM; <cmc)[4-6]). Membranes were extracted from *E. coli* BL21(DE3)pLysS cells that harbour wildtype pET-55-dest-fl-SpsB. Substrate cleavage rates are normalized to DMSO-treated samples from the induced membranes. Background activity from non-induced membranes was subtracted before normalization. The bars highlight the SpsB activity at a 10 µM compound concentration relative to DMSO. Data shown represent mean values ± s.d. of averaged triplicates of n = 2 biologically independent experiments per group (n = 6 for wt); P value: <0.0001 (****); Two-tailed Student's t-test for PK150 versus DMSO-treated groups per condition.

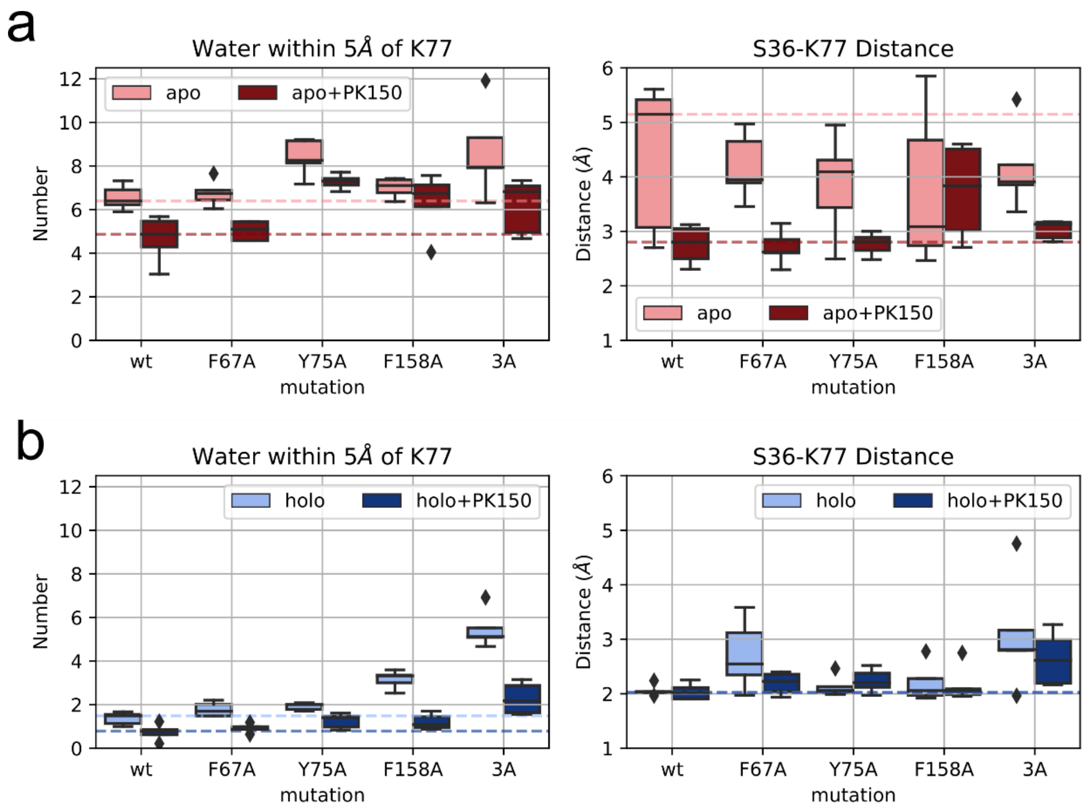

Figure S7. Active site water accessibility and S36-K77 distance of SpsB mutants in (a) apo (light red) and apo+PK150 (dark red) systems and (b) holo (light blue) and holo+PK150 (dark blue) systems. Data points with PK150 RMSD > 15Å are excluded from the analysis. The dashed lines show the average number of water molecules observed in wt SpsB. Data points in the box plot are the mean of each simulation (n=5).

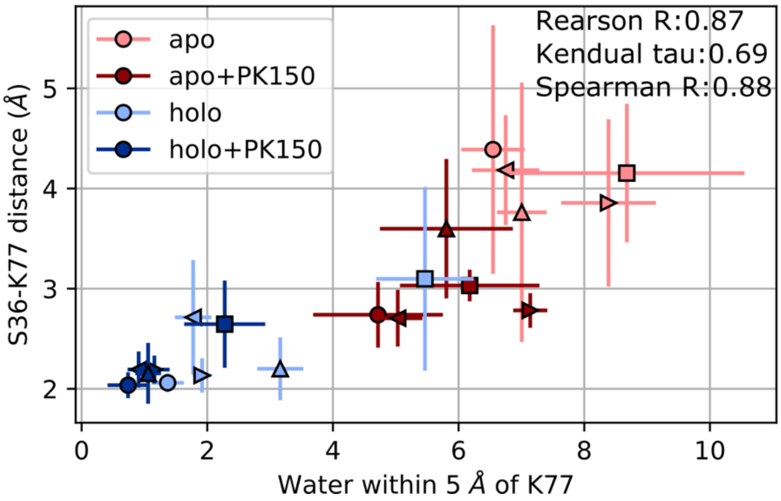

Figure S8. Correlation between the active site water accessibility and the S36-K77 distance in the simulations of wt (circle), F67A (triangle left), Y75A (triangle right), F158A (triangle up), and 3A (square) SpsB mutants. Apo (light red), apo+PK150 (dark red), holo (light blue), and holo+PK150 (dark blue) are depicted in different colors. Data points and error bars show the average values ± s.d across the mean of five independent simulations. Data points with PK150 RMSD > 15Å are excluded from the analysis.

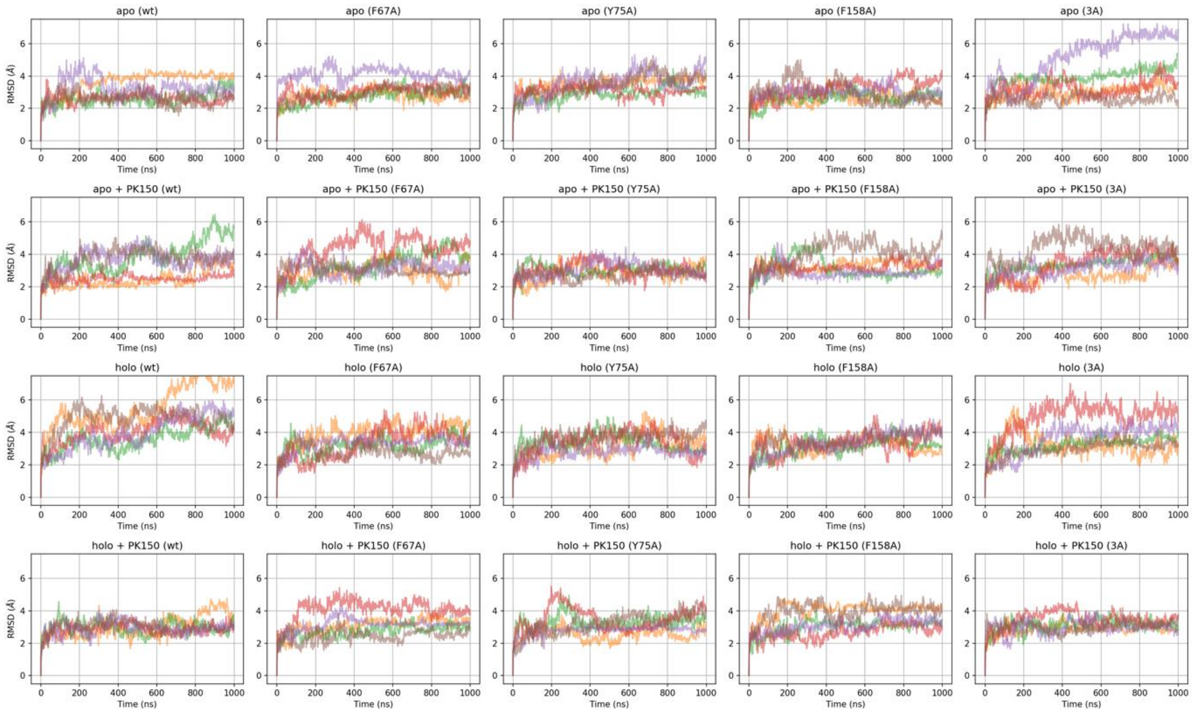

Figure S9. Time evolution of the root-mean-square deviation (RMSD of non-hydrogen atoms) of the SpsB enzymes in the apo, apo+**PK150**, holo, and holo+**PK150** simulations (number of simulations, n= 5, with each simulation represented by a different line color) of wt and mutant SpsB with the enzyme aligned to the starting structure.

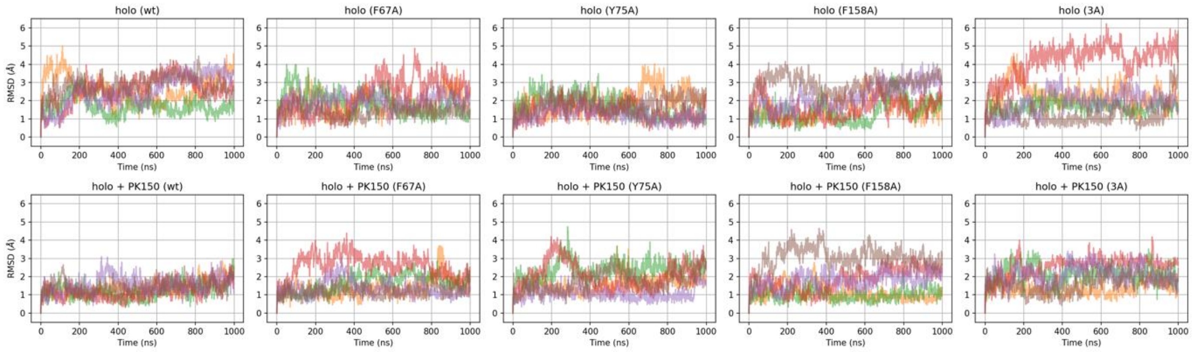

Figure S10. Time evolution of the RMSD of the substrate in the holo and holo+**PK150** simulations of wt and mutant SpsB (n= 5 simulation replicas) after best superposition of the protein on the protein start structure. Note, in this case the RMSD includes not only conformational deviations of the substrate but also overall displacement relative to the protein. Because of the high fluctuation of both termini of the substrate peptide (fully exposed to solvent), only the β-strand region and the scissile bond (TAKKAS) in contact with the protein are used to calculate the RMSD.

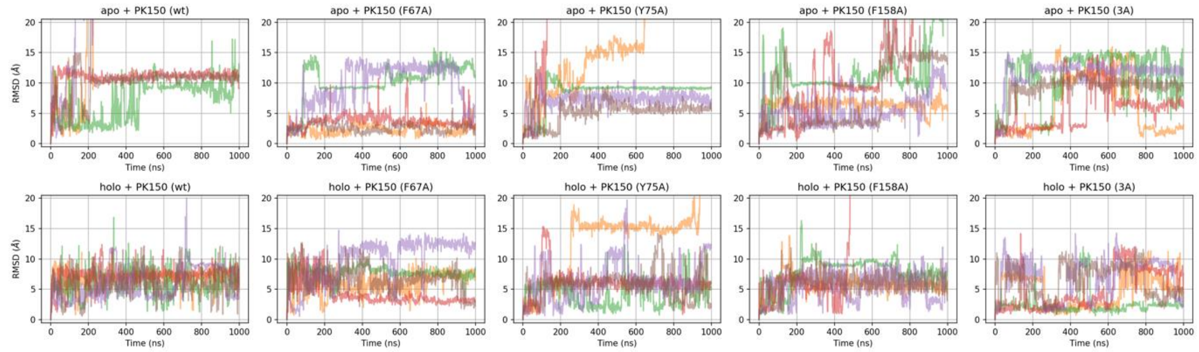

Figure S11. Time evolution of the RMSD of the PK150 ligand in the apo+**PK150** and holo+**PK150** simulations of wt and mutant SpsB (n = 5) after best superposition of the protein on the protein start structure. Note, in this case the RMSD includes not only conformational deviations of the PK150 but also overall displacement relative to the protein (e.g. onset of dissociation). For clarity, the maximum RMSD is capped at 20.5 Å.

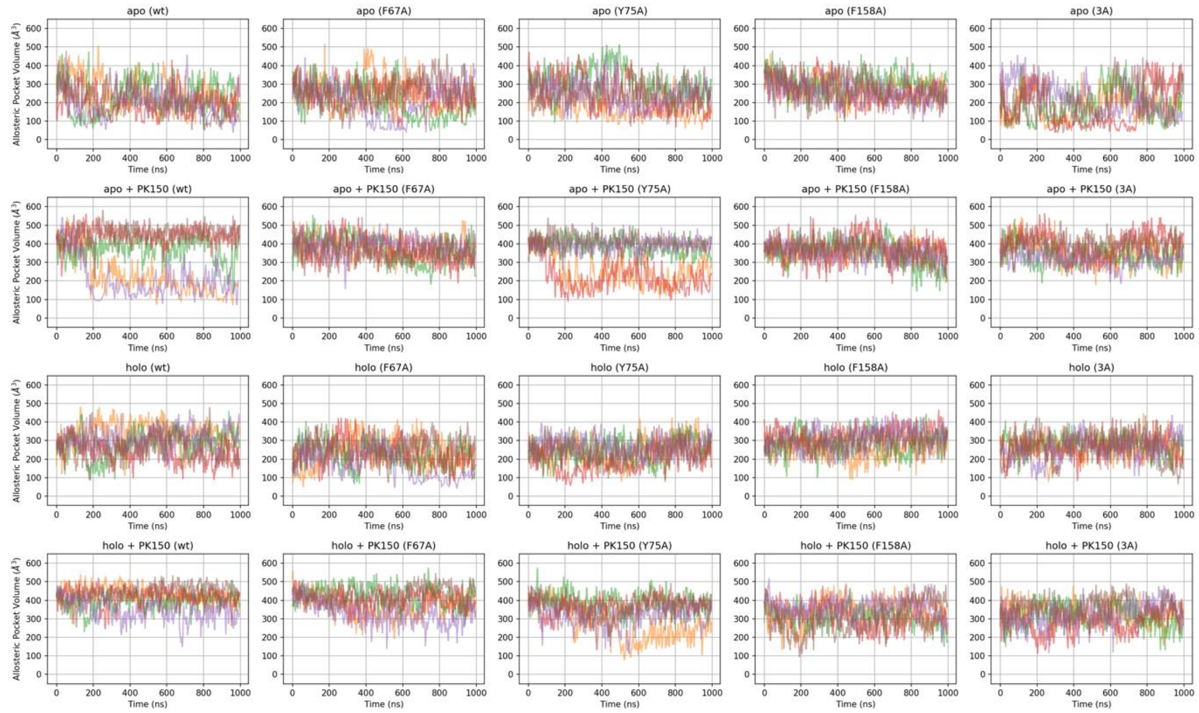

Figure S12. Time evolution of allosteric pocket volume in the apo, apo+**PK150**, holo, and holo+**PK150** simulations of wt and mutant SpsB (n = 5). For clarity the volume is averaged for every 10 ns.

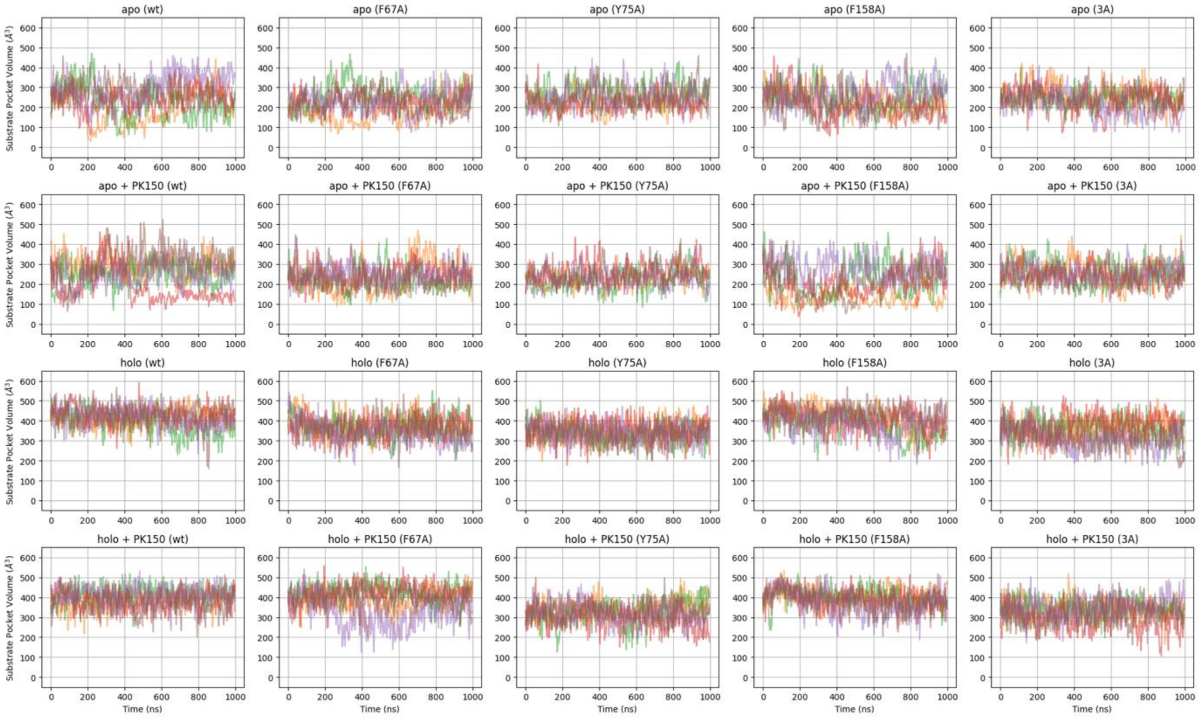

Figure S13. Time evolution of substrate pocket volume in the apo, apo+**PK150**, holo, and holo+**PK150** simulations of wt and mutant SpsB (n = 5). For clarity the volume is averaged for every 10 ns.

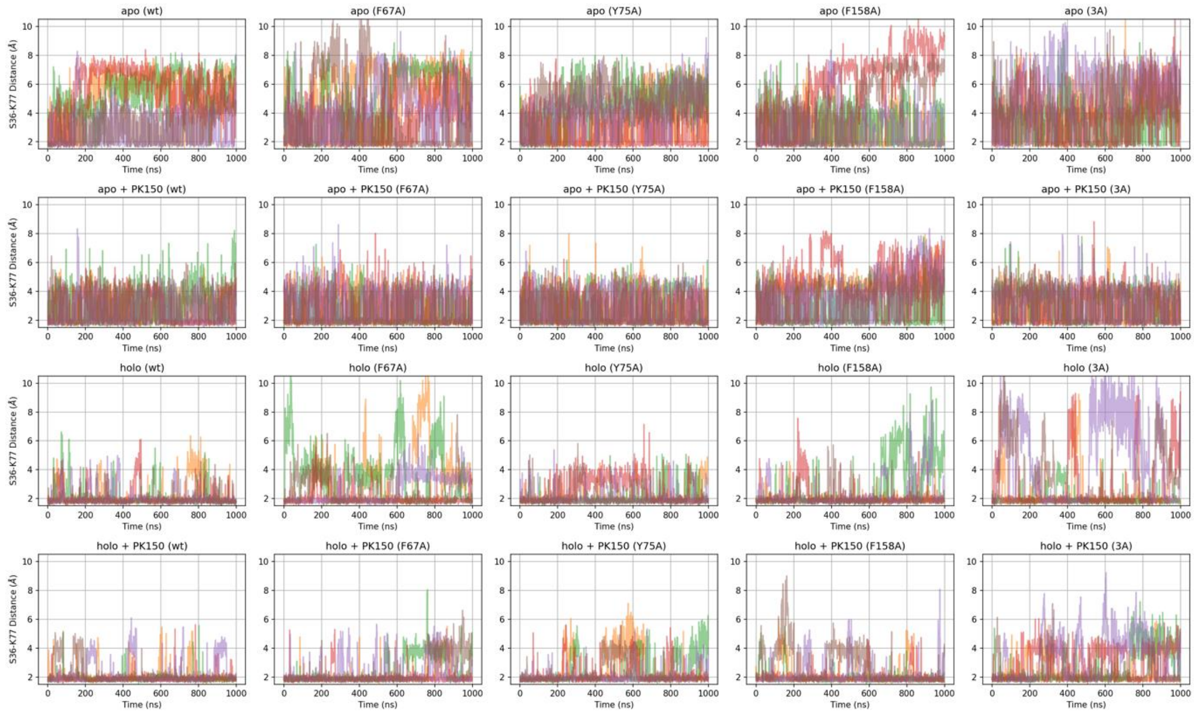

Figure S14. Time evolution of the S36-K77 distance in the apo, apo+**PK150**, holo, and holo+**PK150** simulations of wt and mutant SpsB (n = 5). For clarity, the maximum distance is capped at 10.5 Å.

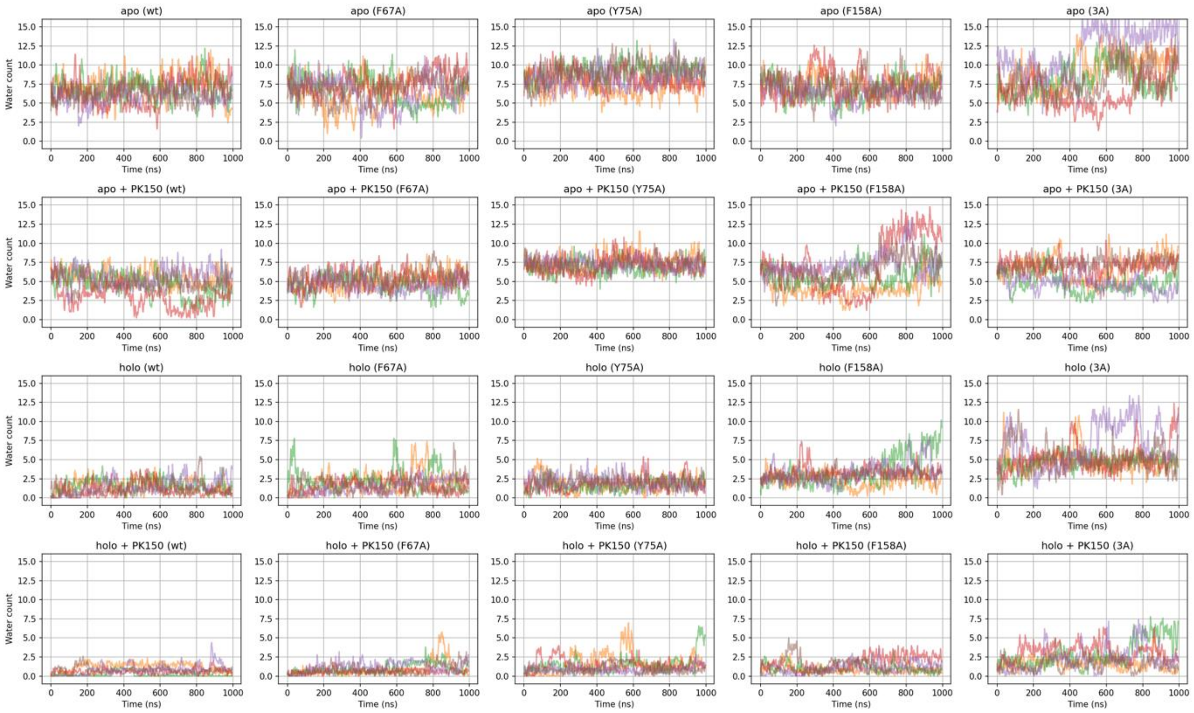

Figure S15. Time evolution of the number of water molecules within 5 Å of K77 in the apo, apo+**PK150**, holo, and holo+**PK150** simulations of wt and mutant SpsB in all simulations (n = 5). For clarity the water count is averaged for every 10 ns and capped at 16.

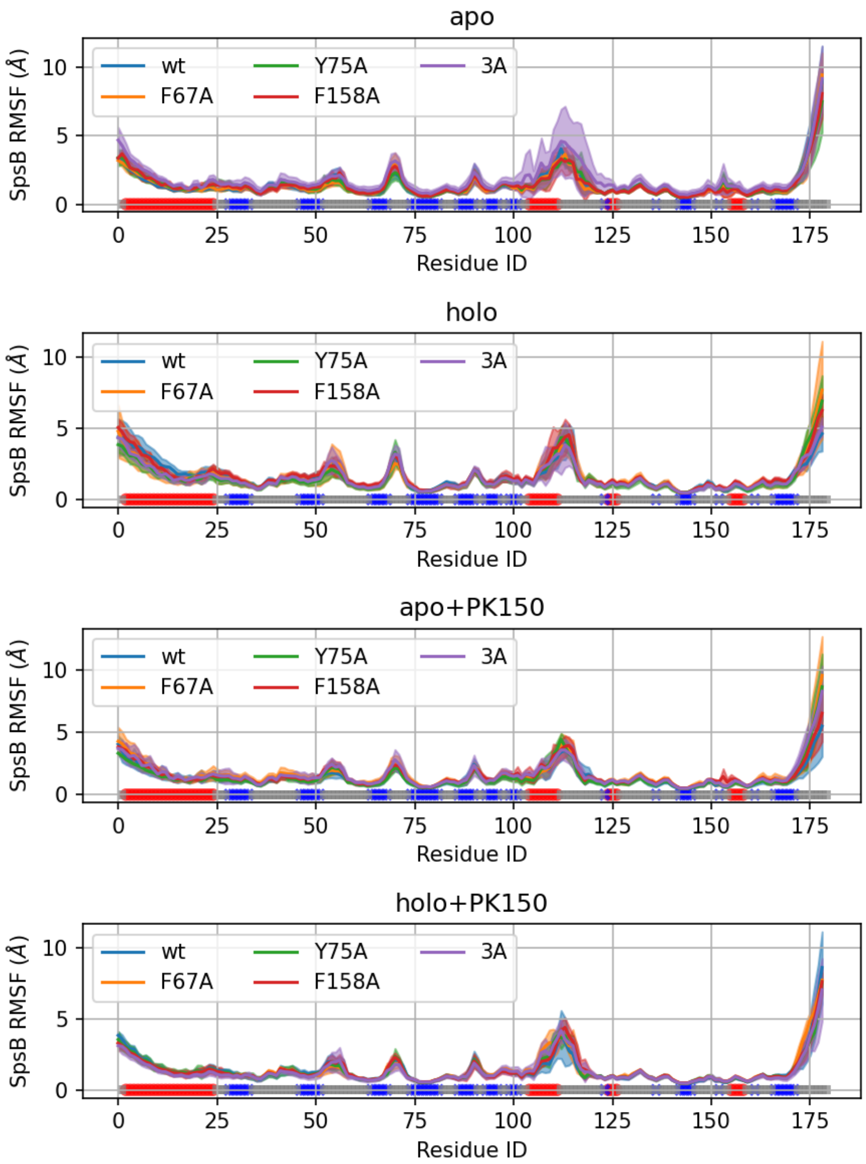

Figure S16. Residue-wise root-mean-square fluctuation (RMSF) of SpsB in the apo, apo+**PK150**, holo, and holo+**PK150** simulations of wt and mutant SpsB (n = 5). The average RMSF is shown together with the standard deviation (shaded area) across five independent simulations.

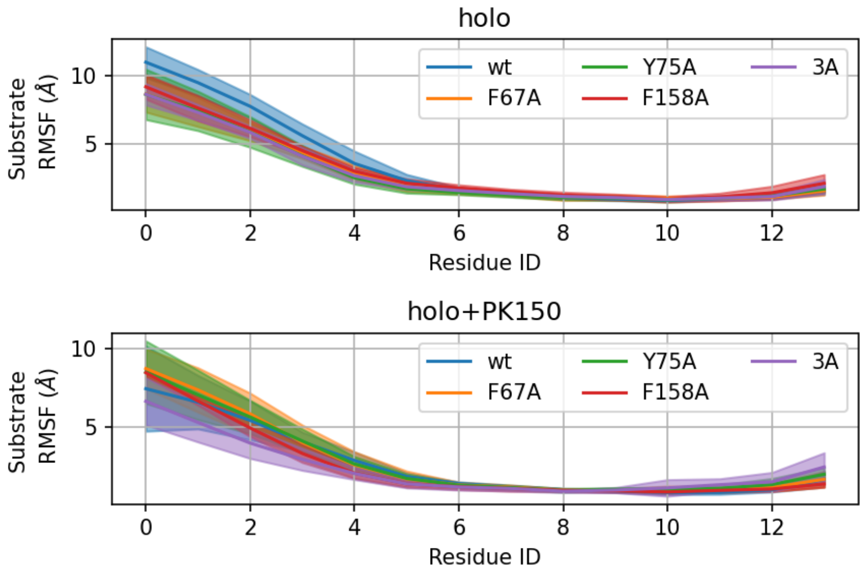

Figure S17. Residue-wise root-mean-square fluctuation (RMSF) of the substrate in the holo and holo+**PK150** simulations of wt and mutant SpsB (n = 5). The average RMSF is shown together with the standard deviation (shaded area) across five independent simulations.

Supplementary Tables S1 – S5

Table S1: Plasmids used in the present work.

Plasmid	Description	Source
**pET-55-dest-fl-SpsB**	attB1, attB2, AmpR, lacI, pUC ori, T7 promotor, Strep-tag II sequence (N-terminal), fl-SpsB cloned into attR1 and attR2 sites of pET-55-DEST, expression clone	Le et al.[7]
**pET-55-dest-fl-SpsB-**<br>**F67A**<br>**Y75A**<br>**F158A**<br>**Q165A**<br>**F67A-Y75A**<br>**F67A-F158A**<br>**FY75A-F158A**<br>**F67A-Y75A-F158A**	attB1, attB2, AmpR, lacI, pUC ori, T7 promotor, Strep-tag II sequence (N-terminal), fl-SpsB with respective point mutations in pET-55-dest, expression clone	This study
**pETMBP-1a-MBP-SpsB**	KanR, lacI, pUC ori, T7 promotor, 6His-Tag, MBP, TEV site, extracellular SpsB domain, expression clone	This study

Table S2: Expected and detected mass shifts obtained from the MSFragger OpenSearch. Detected mass shifts were filtered for shifts >482 Da and mass shift pairs with an exact mass difference of 6.0075 ± 0.0010 Da.

Expected Mass Shift (Da)	Found Mass Shifts (Da)
937.3794 (**PK150-P** + Heavy Tag)	937.3792
931.3719 (**PK150-P** + Light Tag)	931.3724
	744.3952
	742.3716
	738.3646
	736.3744

Table S3: Results modified closed search analysis using FragPipe analysis platform[8-12] for binding site identification of **PK150-P** to SpsB.

Identifier	Modified_Peptide	Log2R replicate 1	Log2R replicate 2	Log2R_Average
Q2FZT7_V66	GNVVV*FHANK	0.090862067	-0.011296246	0.03978291

Table S4: Overview of primer sequences (custom oligos, *Merck*) for QuikChange Site-Directed Mutagenesis of full-length SpsB and order of point mutation introduction for double and triple point mutations.

POINT MUTATION	PRIMER 5’ → 3’ SEQUENCES
**F67A**	FWD 5'-gtttggaaaaaggtaatgtagttgtc**gc**ccatgcaaacaaaaatgatgactatg-3'<br>REV 5’'-catagtcatcatttttgtttgcatgg**gc**gacaactacattacctttttccaaac-3'
**Y75A**	FWD 5’- catgcaaacaaaaatgatgac**gc**tgttaaacgtgtcatcggtgttcc-3’<br>REV 5’- ggaacaccgatgacacgtttaaca**gc**gtcatcatttttgtttgcatg-3’
**F158A**	FWD 5'-aagtaaagatagccgtgcg**gc**tggcctcattgatgaagac-3'<br>REV 5'-gtcttcatcaatgaggcca**gc**cgcacggctatctttactt-3'
**Q165A**	FWD 5’-ggcctcattgatgaagac**gc**aattgttggtaaagtttcatttaggttctg-3’<br>REV 5’-cagaacctaaatgaaactttaccaacaatt**gc**gtcttcatcaatgaggcc-3’
**F67A-Y75A**	1. F67A primers<br>2. Y75A primers
**F67A-F158A**	1. F67A primers<br>2. 158A primers
**Y75A-F158A**	1. 158A primers<br>2. Y75A primers
**F67A-Y75A-F158A**	1. F67A primers<br>2. F158A primers<br>3. Y75A primers

Table S5: Cycling parameters for the QuikChange site-directed mutagenesis PCR reactions.

Time	Temperature	Cycle
**3 min**	98 °C	
**45 s**	95 °C	
**30s**	60 – 68 °C	35 ×
**3 min**	72 °C	
**7 min**	72 °C	
∞	4 °C	

Supporting References

1 Hadkar, U. B. Critical Micelle Concentration of Surfactant Using Hadkar Factor. *Indian J. Pharm. Educ. Res.* **49**, 134-139 (2015).

2 Dai, S. & Tam, K. Isothermal titration calorimetric studies of alkyl phenol ethoxylate surfactants in aqueous solutions. *Colloids Surf. A: Physicochem. Eng. Asp.* **229**, 157-168 (2003).

3 Brown, M. J., Ameer, M. A. & Beier, K. *Vitamin B6 Deficiency*. (StatPearls Publishing, Treasure Island (FL), 2021).

4 Chattopadhyay, A. & Harikumar, K. Dependence of critical micelle concentration of a zwitterionic detergent on ionic strength: implications in receptor solubilization. *FEBS Lett.* **391**, 199-202 (1996).

5 Giacomelli, C. E., Vermeer, A. W. & Norde, W. Micellization and adsorption characteristics of CHAPS. *Langmuir* **16**, 4853-4858 (2000).

6 Qin, X., Liu, M., Yang, D. & Zhang, X. Concentration-Dependent Aggregation of CHAPS Investigated by NMR Spectroscopy. *J. Phys. Chem. B* **114**, 3863-3868 (2010).

7 Le, P. *et al.* Repurposing human kinase inhibitors to create an antibiotic active against drug-resistant Staphylococcus aureus, persisters, and biofilms. *Nat. Chem.* **12**, 145-158 (2020).

8 Kong, A. T., Leprevost, F. V., Avtonomov, D. M., Mellacheruvu, D. & Nesvizhskii, A. I. MSFragger: ultrafast and comprehensive peptide identification in mass spectrometry–based proteomics. *Nat. Methods* **14**, 513-520 (2017).

9 Yu, F. *et al.* Identification of modified peptides using localization-aware open search. *Nat. Commun.* **11**, 4065 (2020).

10 Chang, H.-Y. *et al.* Crystal-C: a computational tool for refinement of open search results. *J. Proteome Res.* **19**, 2511-2515 (2020).

11 Geiszler, D. J. *et al.* PTM-Shepherd: analysis and summarization of post-translational and chemical modifications from open search results. *Mol. Cell. Proteom.* **20** (2021).

12 Teo, G. C., Polasky, D. A., Yu, F. & Nesvizhskii, A. I. Fast deisotoping algorithm and its implementation in the MSFragger search engine. *J. Proteome Res.* **20**, 498-505 (2020).
