# Supplementary material for: Unraveling the mechanism of small molecule induced activation of Staphylococcus aureus signal peptidase IB
Source: Commun Biol. 2024 Jul 24;7:895. doi: 10.1038/s42003-024-06575-x (PMC11266668; doi:10.1038/s42003-024-06575-x)
Supplement: Supplementary file 3 — Description of Additional Supplementary Files [file 42003_2024_6575_MOESM3_ESM.pdf]

Description of Additional Supplementary Files

File name: Supplementary Data 1
Description: Results of binding site data analysis

File name: Supplementary Data 2
Description: Source data of FRET assays
